# iPiDA-GCN: Identification of piRNA-disease associations based on Graph Convolutional Network

**DOI:** 10.1371/journal.pcbi.1010671

**Published:** 2022-10-27

**Authors:** Jialu Hou, Hang Wei, Bin Liu

**Affiliations:** 1 School of Computer Science and Technology, Beijing Institute of Technology, Beijing, China; 2 School of Computer Science and Technology, Xidian University, Xi’an, Shaanxi, China; 3 Advanced Research Institute of Multidisciplinary Science, Beijing Institute of Technology, Beijing, China; University of North Texas, UNITED STATES

## Abstract

**Motivation:**

Piwi-interacting RNAs (piRNAs) play a critical role in the progression of various diseases. Accurately identifying the associations between piRNAs and diseases is important for diagnosing and prognosticating diseases. Although some computational methods have been proposed to detect piRNA-disease associations, it is challenging for these methods to effectively capture nonlinear and complex relationships between piRNAs and diseases because of the limited training data and insufficient association representation.

**Results:**

With the growth of piRNA-disease association data, it is possible to design a more complex machine learning method to solve this problem. In this study, we propose a computational method called iPiDA-GCN for piRNA-disease association identification based on graph convolutional networks (GCNs). The iPiDA-GCN predictor constructs the graphs based on piRNA sequence information, disease semantic information and known piRNA-disease associations. Two GCNs (Asso-GCN and Sim-GCN) are used to extract the features of both piRNAs and diseases by capturing the association patterns from piRNA-disease interaction network and two similarity networks. GCNs can capture complex network structure information from these networks, and learn discriminative features. Finally, the full connection networks and inner production are utilized as the output module to predict piRNA-disease association scores. Experimental results demonstrate that iPiDA-GCN achieves better performance than the other state-of-the-art methods, benefitted from the discriminative features extracted by Asso-GCN and Sim-GCN. The iPiDA-GCN predictor is able to detect new piRNA-disease associations to reveal the potential pathogenesis at the RNA level. The data and source code are available at http://bliulab.net/iPiDA-GCN/.

## Author summary

PiRNAs play critical roles in various biological processes and the abnormal expression of piRNAs may lead to diseases. Meanwhile, several biological experiments show that piRNAs have the potential to be biomarkers or therapeutic targets to diagnose and prognosticate diseases. Some computational methods have been proposed to detect piRNA-disease associations, and provide promising results. However, with the increasing discovery of piRNA-disease associations, the existing methods fail to capture nonlinear and complex association patterns because of the limited training data and insufficient association representation. To overcome above questions, a novel computational method named iPiDA-GCN is proposed for piRNA-disease association identification based on graph convolutional networks. iPiDA-GCN constructs heterogeneous biological networks, and designs Asso-GCN and Sim-GCN modules for learning hidden association patterns in different biological networks. The experimental results show that iPiDA-GCN is able to detect new piRNA-disease associations, and outperforms the other state-of-the-art methods.

This is a *PLOS Computational Biology* Methods paper.

## Introduction

Piwi-interacting RNAs (piRNAs) are a kind of novel small non-coding RNAs (ncRNAs) with 24–35 nucleotides [1,2], often binding to Piwi-subfamily Argonaute proteins [3]. Recently, it is indicated that piRNAs play critical roles in various biological processes by emerging evidences, such as slicing transposable elements in animal’s germline [4], genome defence [5], histone modification [6].

More and more studies have revealed that piRNA abnormal expression leads to many diseases, including cancers, neurodegenerative diseases, geriatric diseases, etc [7]. Several biological experiments show that piRNAs are able to be potential biomarkers or therapeutic targets to diagnose and prognosticate diseases [7,8]. Therefore, it is essential to identify piRNA-disease associations to uncover the pathogenesis of diseases by developing computational methods.

Some databases for piRNA-disease interaction have been constructed. For example, piRDisease v1.0 [9] collects 7939 manually curated associations between 4796 piRNAs and 28 diseases. NcRPheno [10] is a comprehensive ncRNA-disease database containing 1282 experimentally validated piRNA-disease associations. MNDR v3.0 [11] has been proposed to integrate different kinds of ncRNA-disease associations supported by biological literatures, where 13128 piRNA-disease associations between 13365 human piRNAs and 21 diseases are collected. The newly constructed databases store more and more piRNA-disease association information. As a result, the interactions between piRNAs and diseases become sparser and more complex. Therefore, advanced machine learning techniques which are able to fully make use of the available data are the keys to enhance the predictive performance of piRNA-disease association identification.

To reveal the complex interactions between ncRNAs and diseases, several computational methods for ncRNAs-diseases association detection have been proposed. There are mainly three categories of methods for ncRNAs-diseases association detection, including methods based on similarity measure [12,13], methods based on machine learning [14,15] and methods based on network [16]. The research on piRNA-disease association detection is still needed, because the performance of existing computational predictors is still relatively low. Recently, several methods based on machine learning have been proposed for predicting piRNA-disease associations. For example, Wei *et*.*al*. [17] proposed the first piRNA-disease association predictor iPiDA-PUL employing positive unlabeled learning to select negative samples from all unlabeled piRNA-disease associations. With the development of deep learning and its efficiency in processing non-linear data, the deep learning methods are used to extract the features for piRNA-disease predictor [18–20]. For example, the iPiDA-sHN [18] predictor extracted features by Convolutional Neural Network (CNN), and then trained Support Vector Machines (SVMs) with selected high quality negative samples and positive samples. DFL-PiDA [19] based on extreme learning machine model employed a convolutional denoising auto-encoder to extract hidden features. The iPiDA-GBNN predictor [20] based on GrowNet [21] stacked auto-encoder to extract piRNA features.

The existing predictors provide promising results for detecting novel piRNA-disease associations. However, there are still three main problems: (i) With the rapid growth of data, methods based on machine learning showed lower generalization ability and failed to capture more complex and nonlinear relationships between piRNAs and diseases in larger and sparer datasets. (ii) The existing predictors all represent piRNA-disease associations by concatenating piRNA and disease attribute features, ignoring the structure semantic information of biological networks. (iii) The existing deep-learning-based methods treat piRNA-disease association data as Euclidean or grid-like structure data. In fact, the piRNA-disease associations are organized as networks, where the piRNAs or diseases are modelled as vertices, and the associations are viewed as edges. As a result, the existing methods fail to capture complex interactions among piRNA and disease entities, and learn the hidden association patterns in the graph-structured data [22]. To process the complex graph structure data efficiently with deep learning methods, graph convolutional networks (GCNs) [23,24] are proposed to generalize CNN from grid-structured data to graph-structured data, and learn node representations by capturing complex graph structure information and aggregating neighbour node information in the graph. Due to GCN’s powerful ability of capturing complex structure information and potential association patterns, it has also been successfully applied to various tasks in bioinformatics, such as disease-gene association detection [25], drug-target interaction prediction [26,27] and drug repositioning [28].

Inspired by the effectiveness of GCN to capture nonlinear association patterns from complex networks, a novel computational method named iPiDA-GCN is proposed to identify piRNA-disease associations. In particular, two GCN modules are designed to capture the rich semantic information of different biological networks. Asso-GCN module is applied to learn node representations from the piRNA-disease association network, where piRNA node features are learned from associated disease nodes, and disease node features are learned from associated piRNA nodes. Sim-GCN modules are used to further learn the node representations from two homogeneous similarity networks, where piRNA node representations are obtained based on the piRNA neighbour information, and disease node representations are obtained in the same way. Finally, we treat this problem as a link prediction task, and predict the piRNA-disease association scores based on learned features. The experimental results show that iPiDA-GCN outperforms the other state-of-the-art methods, and the visualization of the prediction results further illuminates the advantages of iPiDA-GCN.

## Materials and methods

### Datasets

The comprehensive ncRNA–disease database MNDR v3.0 [11] (http://www.rnadisease.org/) contains the latest and largest piRNA-disease dataset among all the existing piRNA-disease databases. The human piRNAs with sequence information are extracted from piRBase v3.0 (http://bigdata.ibp.ac.cn/piRBase/) [29]. After removing duplicate associations, 11981 experimentally verified piRNA-disease associations containing 10149 piRNAs and 19 diseases are collected. The datasets are represented as:

$$\left\{ \begin{array}{c} S_{all}=S_{independent}+S_{benchmark}\\ S_{all}=S_{all}^{+}\cup S_{all}^{-}\\ S_{benchmark}=S_{train}+S_{validation} \end{array}\right.$$ (1)

where the dataset $S_{all}$ is divided into a benchmark set $S_{benchmark}$ and an independent test set $S_{independent}$. $S_{all}^{+}$ represents the positive set containing 11981 positive associations and $S_{all}^{-}$ represents the negative set containing 180850 negative associations. The benchmark set $S_{benchmark}$ is randomly divided into five subsets, where four subsets are considered as the training set $S_{train}$, and the remaining one is used as the validation set $S_{validation}$. The hyperparameters of our method are optimized on the validation set via five-fold cross validation. The influence of hyperparameters on the performance of iPiDA-GCN is shown in S1 **Supplementary Material**

Finally, the model is evaluated on the independent test set $S_{independent}$ to compare with the other related methods.

### Method overview

**Fig 1:**
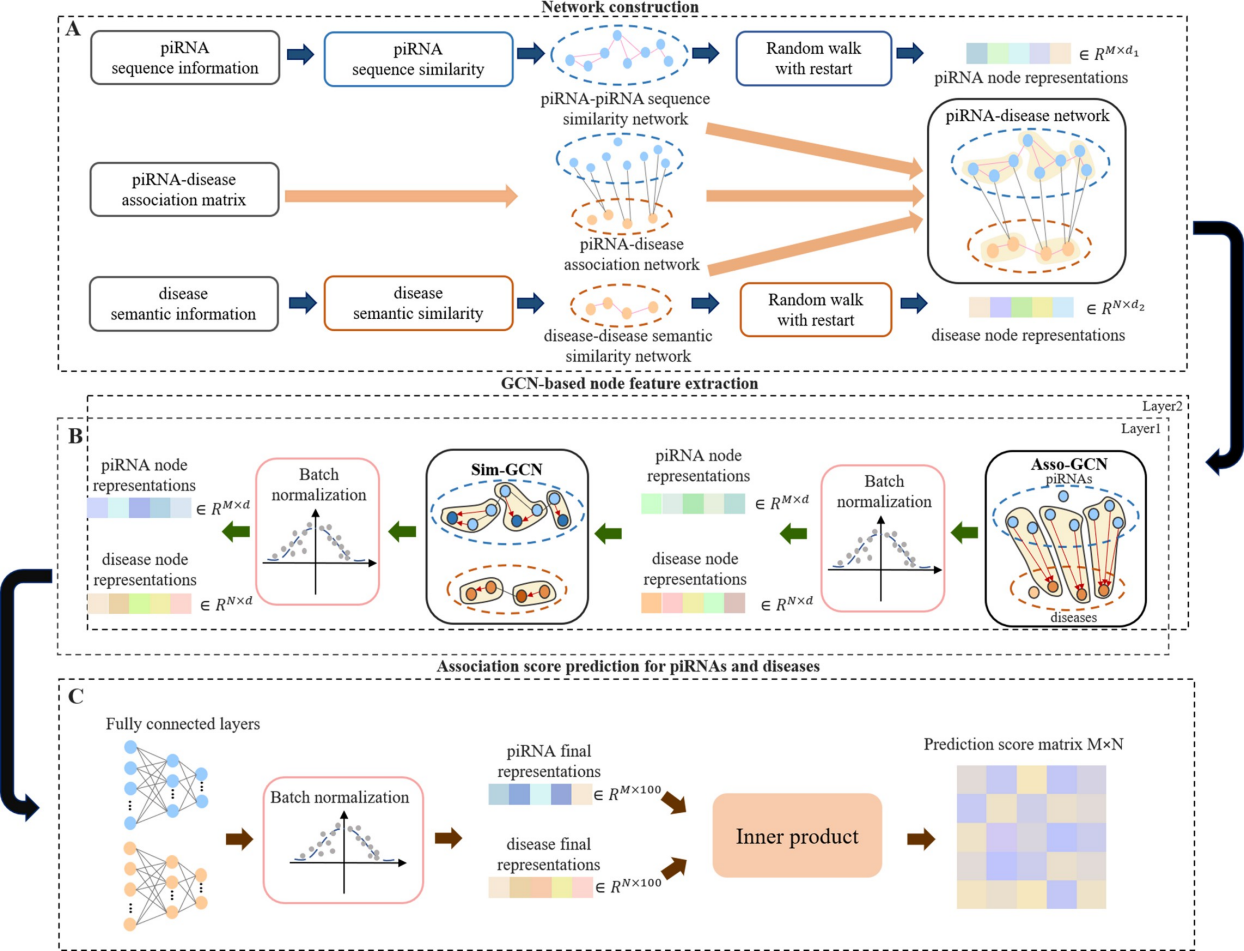
The flowchart of iPiDA-GCN. iPiDA-GCN mainly contains three modules: (i) Heterogeneous network construction (**Fig 1A**). Three kinds of edges are collected in the heterogeneous piRNA-disease association network, including piRNA-piRNA similarities, disease-disease similarities and piRNA-disease interactions. (ii) GCN-based node feature extraction (**Fig 1B**). Asso-GCN and Sim-GCN modules are designed to continuously learn node features from different subnetworks of piRNA-disease association network. Specifically, Asso-GCN captures hidden associated features of heterogeneous nodes from piRNA-disease interaction subnetwork, while Sim-GCN captures hidden associated features of homogeneous nodes from two similarity subnetworks. (iii) Association prediction for piRNAs and diseases (**Fig 1C**). Three fully connected layers are employed to learn the low-dimensional representations of piRNAs and diseases. Finally, association scores between piRNAs and diseases are computed through inner product operation.

In this section, we propose a predictor iPiDA-GCN based on GCN to predict piRNA-disease associations. The framework of iPiDA-GCN is shown in **Fig 1**, it mainly contains three steps: heterogeneous network construction (**Fig 1A**), GCN-based node feature extraction (**Fig 1B**) and association prediction for piRNAs and diseases (**Fig 1C**).

### Network construction

#### Edge representation

There are three types of edges in the constructed piRNA-disease network. One type of the edges is the original interactions between *m* piRNAs (*m* = 10149) and *n* diseases (*n* = 19). The piRNA-disease association adjacency matrix is represented as **A**_PD_ in **Eq 2**, where *a*_*i*,*j*_ = 1 if the *i-*th piRNA is associated with the *j-*th disease with experimental verification, otherwise *a*_*i*,*j*_ = 0.


$$A_{\mathrm{PD}}=[\begin{array}{cccc} a_{1,1} & a_{1,2} & \ldots & a_{1,n}\\ a_{2,1} & a_{2,2} & \cdots & a_{2,n}\\ \vdots \vdots & \ddots & \vdots & \\ a_{m,1} & a_{m,2} & \cdots & a_{m,n} \end{array}]$$ (2)

The other two types of edges named ‘similarity edge’ are contained in the similarity subnetworks, and calculated based on the biological entities’ information. Specifically, piRNA-piRNA similarity **S**_*p*_ is obtained based on piRNA sequence information, downloaded from piRBase v3.0 [29]. The sequence information contains the attribute information of non-coding RNAs, and the Smith-Waterman alignment algorithm [30] can effectively capture the functional similarities among RNAs. In this study, the piRNA sequence similarity **S**_*p*_(*p*_*i*_, *p*_*k*_) between *i-*th piRNA *p*_*i*_ and *k-*th piRNA *p*_*k*_ is computed as:

$$S_{p}(p_{i},p_{k})=\frac{SW(p_{i},p_{k})}{\sqrt{SW(p_{i},p_{i})\times SW(p_{k},p_{k})}}$$ (3)

where the SW(*p*_*i*_, *p*_*k*_) is the sequence alignment value between the *i-*th piRNA and *k-*th piRNA calculated by the Smith-Waterman alignment algorithm [30].

Disease-disease similarity is computed based on disease ontology (DO) [31], which is used as a standard representation of human disease in biomedical ontologies [32]. DO is capable of translating molecular findings from high-throughput data to clinical relevance. DOSE [33] provides different semantic similarity algorithms based on DO terms. The algorithm based on Directed Acyclic Graph (DAG) [34] has been widely used in ncRNA-disease association detection [35–37]. It cannot only provide consistent semantic similarities, but also can detect potential relations between complex diseases. Therefore, the disease semantic similarity between disease *d*_*k*_ and disease *d*_*j*_ is be calculated as [34]:

$$S_{d}(d_{k},d_{j})=\frac{\sum_{t\in T_{k}⋂T_{j}}\left(S_{d_{k}}(t)+S_{d_{j}}(t)\right)}{\sum_{t\in T_{k}}S_{d_{k}}(t)+\sum_{t\in T_{j}}S_{d_{j}}(t)}$$ (4)

where T_*k*_ is the set containing all diseases in the DAG of disease *d*_*k*_, and $S_{d_{k}}(t)$ denotes the semantic contribution of disease *t*∈T_*k*_ to the *k-*th disease calculated by [34]:

$$\left\{ \begin{array}{ll} S_{d_{k}}(t)=max\{\alpha *S_{d_{k}}(t\prime )|t^{\prime }\in \mathrm{children}\;\mathrm{of}(t)\} & if\;d_{k}\neq d_{j}\\ S_{d_{k}}(t)=1 & otherwise \end{array}\right.$$ (5)

where *α* is the semantic contribution factor set as 0.5 following [34]. The farther the distance between disease *t* and its ancestor is, the lower the semantic contribution of disease *t* to disease *d*_*k*_ is.

#### Node representation

There are two types of nodes representing piRNAs and diseases in the constructed heterogeneous piRNA-disease association network. In this study, random walk with restart (RWR) [38] is employed to optimize the connectivity relationships among the same biological entities, especially for the non-neighbouring and higher-order nodes, and then the optimized similarity matrices are used as the initial feature matrices. The piRNA sequence similarity matrix and disease semantic similarity matrix are used as the input of RWR. The initial node features can be obtained by considering the global topology information of each network. The piRNA node representation generated by RWR is calculated as [38]:

$$P_{i,j}^{k+1}(i)=(1-\alpha )e_{i,j}+\alpha P_{i,j}^{k}(i)S_{p}(p_{i},p_{j})$$ (6)

$$P(i)=[P_{i,1}^{\infty }(i),P_{i,2}^{\infty }(i),\ldots ,P_{i,j}^{\infty }(i),{\ldots ,P}_{i,m}^{\infty }(i)]$$ (7)

where $P_{i,j}^{k}(i)$ denotes the probability of walking from piRNA node *p*_*i*_ to node *p*_*j*_ after *k* steps. *e*_*i*,*j*_ denotes the initial probability of walking from piRNA node *p*_*i*_ to node *p*_*j*_, which is the element of an identity matrix. **S**_*p*_(*p*_*i*_, *p*_*j*_) denotes the transition probability obtained from similarity matrix **S**_*p*_, *α* is the restart probabilities. The probability of *p*_*i*_ associated with all the other piRNA nodes are concatenated to generate the node representation **P**(*i*) for piRNA *p*_*i*_. Similarly, the disease node representation **D**(*i*) can be represented as [38]:

$$D_{i,j}^{k+1}(i)=(1-\alpha )e_{i,j}+\alpha D_{i,j}^{k}(i)S_{d(d_{i},d_{j})}$$ (8)

$$D(i)=[D_{i,1}^{\infty }(i),D_{i,2}^{\infty }(i),\ldots ,D_{i,j}^{\infty }(i),{\ldots ,D}_{i,n}^{\infty }(i)]$$ (9)

To overcome the problem of insufficient representation, the feature dimension of each disease node is further extended from 19 to 1000 with polynomial features derived from the original input features, which can better reflect the interactions of different features in different dimensions. Specifically, polynomial features denote the polynomial combinations of the features with degree less than or equal to the specified degree. For example, given a disease node represented by the semantic similarities with three diseases *a*, *b* and *c*, it can be extended by degree-2 polynomial features as $\left[a,b,c,a^{2},a\times b,a\times c,b^{2},b\times c,c^{2}\right]$.

### GCN-based node feature extraction

The key step of identifying piRNA-disease associations is node representation based on Graph Convolution Network (GCN). GCN can aggregate neighbour node information, and capture the hidden network structures to powerfully extract discriminative node features. Therefore, we employ GCN to learn the features of piRNA and disease nodes from the heterogeneous piRNA-disease association network.

Let **H**^*l*^∈R^*d*^ denotes the node embedding of *l*-th GCN layer, the node embedding **H**^*l*+1^∈R^*d*^ is computed by (*l*+1)-th GCN layer according to [24] as:

$$H^{l+1}=\sigma ({\tilde{D}}^{-\frac{1}{2}}\tilde{S}{\tilde{D}}^{-\frac{1}{2}}H^{l}W^{l})$$ (10)

$$\tilde{S}=I+S$$ (11)

$$\tilde{D}(i,i)=\sum_{j}\tilde{S}(i,j)$$ (12)

where **S** is the adjacency matrix denoting the relationships among all nodes in the network, and **I** is an identity matrix. $\tilde{D}$ represents the degree matrix of $\tilde{S}$, **W**^***l***^ denotes the trainable parameter matrix of GCN model, *σ*(∙) is a nonlinear activation function.

Two main modules Asso-GCN and Sim-GCN are designed to extract node representation. As shown in **Fig 1B,** Asso-GCN is adopted to aggregate node information from the piRNA-disease interaction network, G_*asso*_ = {V_*p*_, V_*d*_, E_*p−d*_} where *V*_*p*_ and *V*_*d*_ represent piRNA and disease nodes respectively, while E_*p−d*_ represents the interactions between piRNA and disease nodes. The piRNA node features are captured from neighbor disease node information and vice versa. Secondly, Sim-GCN is further used to capture semantic information from two different homogeneous similarity networks. The node representations obtained by Asso-GCN are viewed as initial node features in Sim-GCN module. The constructed piRNA-piRNA similarity network G_*p*_ = {V_*p*_, E_*p−p*_} and disease-disease similarity network *G*_*d*_ = {V_*d*_, E_*d−d*_} are two main inputs for Sim-GCN. PiRNA node representations are generated by capturing neighbor piRNA information and disease node representations are learned from neighbor disease information. It is worth noting that batch normalization [39] is conducted following each deep learning module so as to reduce internal covariate shift and increase stability.

The reasons why we designed Asso-GCN and Sim-GCN modules to learn node representations in turn are as followings: In Asso-GCN module, GCN captures node information from the bi-partite piRNA-disease graph, where node representations are only learned from their heterogeneous neighbor nodes. However, piRNA-disease associations are too sparse to provide enough information for GCN to capture discriminative representations. Therefore, we introduce side information, including disease semantic similarity and piRNA sequence similarity. Then, we performed Sim-GCN on the similarity networks to fine-tune the node representations by their homogeneous neighbor nodes.

### Association prediction for piRNAs and diseases

To further eliminate redundancy and noise, three consecutive fully connected layers are designed to extract high-level node features. There are 400, 200 and 100 neurons in each layer. Given a piRNA node representation $h_{p_{i}}$ and a disease node representation $h_{d_{j}}$ extracted from GCN modules, the final piRNA and disease node representation $h_{p_{i}}^{\prime }$ and $h_{d_{j}}^{\prime }$ can be obtained through the dense operation. Then the association score between piRNA *p*_*i*_ and disease *d*_*j*_ is calculated as:

$$U_{i,j}=h_{p_{i}}^{\prime }{h_{d_{j}}^{\prime }}^{T}$$ (13)

where **U** is the final prediction score matrix. The higher the element **U**_*i*,*j*_ is, the more likely piRNA *p*_*i*_ is associated with disease *d*_*j*_.

The mean square error is adopted as the loss function to minimize the Frobenius norm of the difference between predicted score matrix **U** and label matrix **A**_PD_. However, the number of negative associations is much more than that of positive associations. In order to alleviate the imbalance of training samples, *α*-enhanced loss function [25,40] focusing on positive sample learning is used, and can be formulated as:

$$Loss={\left\|{\tilde{A}}_{PD}-U\right\|}_{F}^{2}+\mu {\left\|W\right\|}_{2}^{2}$$ (14)

where

$${\tilde{A}}_{PD}=\left\{ \begin{array}{c} 0\;\mathrm{if}\;A_{PD}(i,j)=0\;\mathrm{or}\;A_{PD}(i,j)\in S_{independent}\\ \alpha \;\mathrm{otherwise} \end{array}\right.$$ (15)

${\tilde{A}}_{PD}$ is the enhanced association matrix generated based on the original adjacency matrix **A**_PD_. *α* is a hyper parameter controlling the margin between true labels and predicted scores. *μ* is a decay factor regulating all trainable model parameters **W**. **U** is the predicted score matrix predicted by iPiDA-GCN.

### Performance evaluation

PiRNA-disease association identification can be viewed as a link prediction task. Two widely used evaluation metrics, including AUC (area under the receiver operating characteristics curve) and AUPR (area under the precision recall curve) [41,42] are used to measure the performance of different methods. The higher AUC and AUPR are, the better the performance of the method is [43].

## Results and discussion

### The effect of GCN layers

GCN is the key module of iPiDA-GCN, which can aggregate information from neighbor nodes and obtain representations of piRNAs and diseases. The number of GCN layers has an important impact on the predictive performance. The influence of the different number of GCN layers is shown in **Table 1**, from which we can see the followings: (i) iPiDA-GCN turns to approximately randomly guess without using GCN module (layers = 0), where the input features are directly processed by fully connected layers and inner production. It achieves better performance when using GCN to capture the potential network structures. The reason is that limited GCN layers cannot capture enough structural information, while stacking more GCN layers can expand the receptive field with aggregating high-order connected node information to obtain expressive representations. (ii) When more layers are added, the performance of iPiDA-GCN gradually increases in terms of both AUC and AUPR, but its performance decreases when more than two layers are added. The reason is that more layers may introduce more noise and irrelevant information into node representation learning, leading to over-smoothing and performance decrement [44,45]. We conclude that GCN with two layers cannot only capture the complex interaction patterns, but also incorporate the node attribute features for representation learning so as to enhance the predictive ability.

**Table 1:** The impact of GCN layers on the predictive performance of iPiDA-GCN on $S_{benchmark}$.

Number of Layers	AUC	AUPR
0	0.5373	0.5328
1	0.6461	0.6320
**2**	**0.6822**	**0.6620**
3	0.6785	0.6566
4	0.6782	0.6606

### Impact of the different components on the performance of iPiDA-GCN

There are three main components in iPiDA-GCN for extracting node features, including a fully connected network, Asso-GCN and Sim-GCN. To analyze the contributions of different components in iPiDA-GCN, three comparative baseline predictors (iPiDA-FN, iPiDA-AssoGCN and iPiDA-SimGCN) are constructed, where iPiDA-FN is constructed only by a fully connected network, while iPiDA-AssoGCN and iPiDA-SimGCN are constructed by combining different GCN modules and fully connected networks. Their performance along with iPiDA-GCN is shown in **Table 2**, from which we can see the followings: (i) Compared with iPiDA-FN, two predictors based on GCN modules achieve much better performance, indicating that GCN contributes to node representations; (ii) Sim-GCN plays a more important role than Asso-GCN for capturing semantic information from two similarity networks; (iii) iPiDA-GCN is superior to all the other baseline predictors, indicating that different components are complementary and contribute to extracting high-level node features, leading to performance improvement.

**Table 2:** The performance of iPiDA-FN, iPiDA-AssoGCN, iPiDA-SimGCN and iPiDA-GCN on $S_{independent}$.

Method	AUC	AUPR
iPiDA-FN	0.5291	0.5107
iPiDA-AssoGCN	0.5603	0.5767
iPiDA-SimGCN	0.6765	0.6559
iPiDA-GCN	**0.7149**	**0.7036**

### Performance comparison among different methods

To demonstrate the effectiveness of iPiDA-GCN, three state-of-the-art predictors are compared, including iPiDA-PUL [17], iPiDA-sHN [18] and piRDA [46]. All these three predictors have released the source codes or constructed the web servers, facilitating fair performance comparison. Furthermore, in order to evaluate the impact of different learning node representations on the performance of piRNA-disease association prediction, a predictor iPiDA-DW based on the node representation algorithm DeepWalk [47] is also compared with our method, which performs on the heterogeneous piRNA-disease network ignoring node attribute information, and computes association scores followed by full connection networks and inner production. The results of various methods on $S_{independent}$ are listed in **Table 3**, from which we can see the followings: (i) iPiDA-GCN achieves the best performance; (ii) Compared with the methods based on node attribute iPiDA-PUL [17], iPiDA-sHN [18] and piRDA [46], iPiDA-GCN is able to capture hidden structural features, leading to better performance; (iii) Compared with iPiDA-DW which is a method based on network embedding, iPiDA-GCN cannot only incorporate hidden structural and attribute features, but also can learn discriminative node representations through two-level GCNs.

**Table 3:** Performance comparison among different methods on $S_{independent}$.

Method	AUC	AUPR
iPiDi-PUL^a^	0.6653	0.6550
iPiDA-sHN^b^	0.5226	0.5203
iPiDA-DW^c^	0.6317	0.6211
piRDA^d^	0.4939	0.5116
iPiDA-GCN^e^	**0.7149**	**0.7036**

^a^ Results obtained by reproducing the iPiDi-PUL predictor with the help of its source code with parameters (n_components = 200, n_estimators = 150, max_features = 0.2, number of ensemble learner = 5)

^b^ Results obtained by reproducing the iPiDA-sHN predictor with the help of its source code with parameters (C = 1.0, kernel = ‘rbf’, gamma = 1)

^c^ The parameters number-walks = 10, walk-length = 80, window-size = 10

^d^ The results are generated with the help of the web server of piRDA (http://nsclbio.jbnu.ac.kr/tools/piRDA/). Because piRDA is constructed based on an outdated dataset, it can only predict the piRNAs associated with 13 diseases in $S_{independent}$. Therefore, only the prediction results for these associations are evaluated

^e^ The parameters epoch = 2000, learning rate = 0.001, weight decay factor = 1.0.

### Visualization of predicted associations by iPiDA-GCN

**Fig 2:**
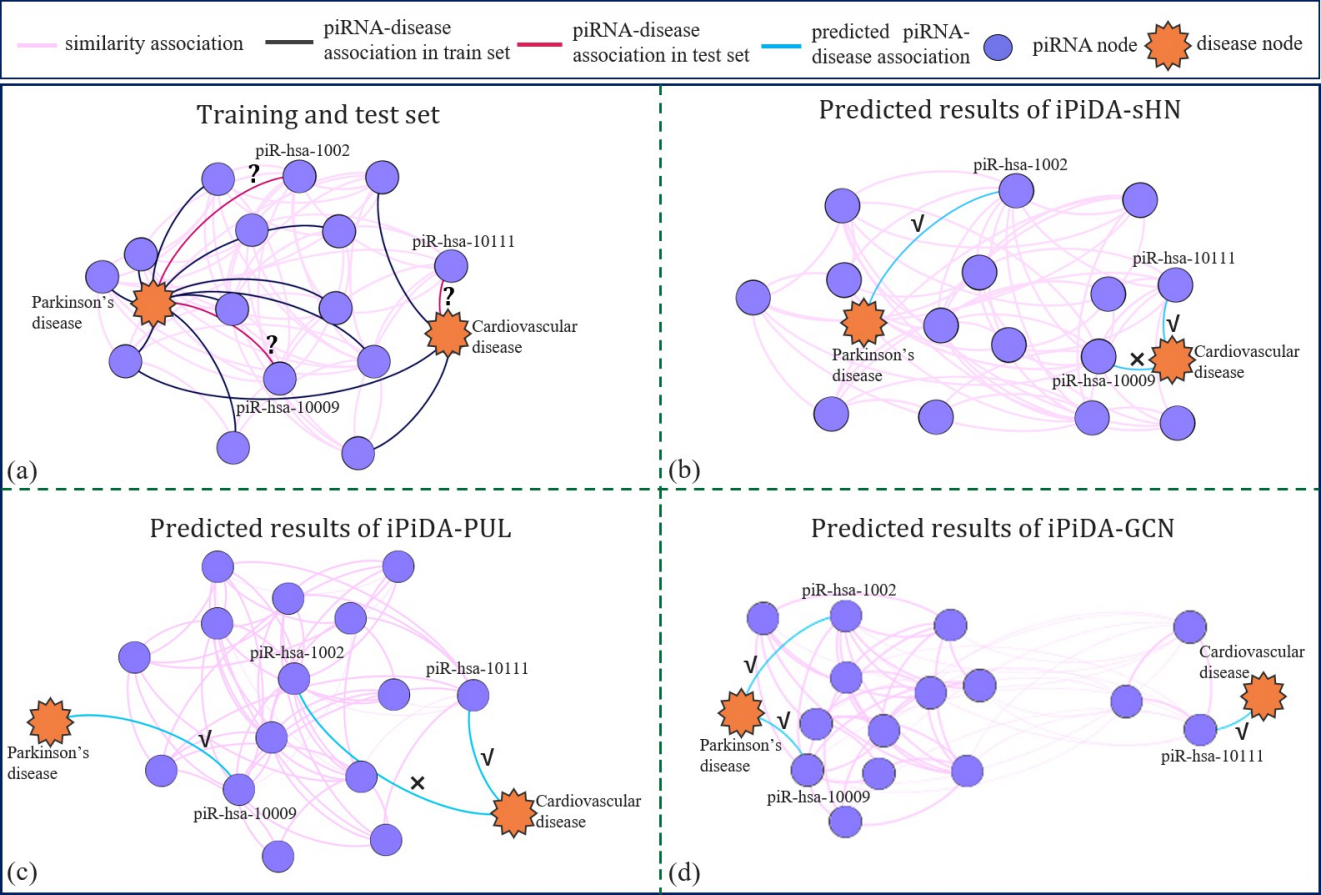
The prediction visualization of iPiDA-GCN and compared methods. These figures are plotted with the help of Gephi [49]. The nodes shown in orange and purple represent diseases and piRNAs, respectively. Pink lines denote the similarity associations between piRNAs, and black and red lines denote piRNA-disease associations in the training set and test set, respectively. The piRNA-disease associations predicted by different models are represented by blue lines.

In order to explore why iPiDA-GCN is able to accurately predict the potential associations between piRNAs and diseases, the prediction results of three piRNA-disease associations in the test set (<piR-has-1002, Parkinson’s disease>, <piR-has-10009, Parkinson’s disease> and <piR-has-10111, Cardiovascular disease>) are selected, and visualized in **Fig 2**, from which we can see the followings: (i) iPiDA-PUL [17] and iPiDA-sHN [18] predict that piR-has-1002 and piR-has-10009 are associated with cardiovascular disease without experimental verification. iPiDA-PUL is a discriminative model based on manually constructed features, failing to learn complex association patterns. iPiDA-sHN adopts CNN to extract node features, but CNN is not suitable for analyzing the graph-structured piRNA-disease association data [48]. (ii) iPiDA-GCN correctly predicts the piRNA-disease associations in the test set, owing to its informative features learned by aggregating graph structure information from the complex piRNA-disease network. Therefore, iPiDA-GCN outperforms the other existing methods for predicting these three piRNA-disease associations.

### Case study

In order to evaluate the performance of iPiDA-GCN for identifying piRNAs associated with known diseases, four important and major diseases (“Cardiovascular disease”, “Renal cell carcinoma”, “Alzheimer’s disease” and “Parkinson’s disease”) are selected and their associated piRNAs are predicted by using iPiDA-GCN. **Table 4** lists the top 5 predicted piRNAs for each disease. It can be seen from **Table 4** that 19 of the 20 predicted piRNA-disease associations have been verified by the biological literatures. For example, piR-hsa-31280 is down-regulated in cardiovascular disease tissues [50]. piR-hsa-8245 is up-regulated in cardiovascular disease tissue and has a higher expression about 5-fold in cardio sphere (CS) compared with cardio-sphere-derived cells (CDC) [50]. piR-hsa-10732 shows down-regulation in renal cell carcinoma tissue [51]. The expression of piR-hsa-28131 is different in Alzheimer’s disease-affected brain compared with the normal human brain [3]. In addition, the top five identified piRNAs associated with Parkinson’s disease (PD) are differently regulated in cells between control and PD-patients [52]. The prediction results show that iPiDA-GCN can discover new potential piRNA-disease associations, where the unconfirmed associations can be viewed as candidates to provide guidance for biological experiments in the future.

**Table 4:** The top 5 piRNAs associated with different diseases predicted by iPiDA-GCN.

Disease	Rank	piRNA	Evidence^a^
Cardiovascular disease	1	piR-hsa-1191	PMID:27131603
	2	piR-hsa-31280	PMID:27131603
	3	piR-hsa-8245	PMID:27131603
	4	piR-hsa-18089	PMID:27131603
	5	piR-hsa-27115	PMID:27131603
Renal cell carcinoma	1	piR-hsa-10732	PMID:26071182
	2	piR-hsa-29578	PMID:26071182
	3	piR-hsa-9186	PMID:26071182
	4	piR-hsa-19501	PMID:26071182
	5	piR-hsa-3161	PMID:26071182
Alzheimer’s disease	1	piR-hsa-28131	PMID:28127595
	2	piR-hsa-2107	PMID:28127595
	3	piR-hsa-1207	PMID:28127595
	4	piR-hsa-12790	Unconfirmed
	5	piR-hsa-2106	PMID:26934981
Parkinson’s disease	1	piR-hsa-356	PMID:29986767
	2	piR-hsa-6015	PMID:29986767
	3	piR-hsa-5249	PMID:29986767
	4	piR-hsa-24512	PMID:29986767
	5	piR-hsa-10122	PMID:29986767

^a^ The detected piRNA-disease associations are validated by the biological literatures in PubMed. The PMIDs of these literatures are listed.

## Conclusion

In this study, we propose a novel computational method called iPiDA-GCN to identify piRNA-disease associations based on graph convolutional networks. Experimental results show that iPiDA-GCN is superior to the other state-of-the-art methods. Three main factors attribute to the superior performance of iPiDA-GCN: (i) Multiple biological data sources are used to construct the heterogonous piRNA-disease association network, covering more informative interactions among biological entities; (ii) Asso-GCN and Sim-GCN modules are designed to reasonably capture the graph structure information and hidden association patterns; (iii) iPiDA-GCN obtains final piRNA and disease features with three fully connected networks, which is able to filter noise, and extract meaningful information.

Besides, although iPiDA-GCN is designed for piRNA-disease association detection, it has the potential to be extend to other biological link prediction tasks, such as protein-protein interaction prediction [53], RNA-gene interaction detection [54,55].

## Supporting information

S1 Supplementary Material The hyper-parameters of GCN in iPiDA-GCN. (PDF)
