# Supplementary material for: iPiDA-GCN: Identification of piRNA-disease associations based on Graph Convolutional Network
Source: PLoS Comput Biol. 2022 Oct 27;18(10):e1010671. doi: 10.1371/journal.pcbi.1010671 (PMC9662734; doi:10.1371/journal.pcbi.1010671)
Supplement: S1 Supplementary Material — (PDF) [file pcbi.1010671.s001.pdf]

Supplementary material

Three hyper-parameters are analyzed to uncover their influence on the performance of our model, including epoch, learning rate and weight decay factor. All the hyper-parameter tuning experiments are performed on the benchmark dataset via five-fold cross-validation. We analyze the impact of parameters by varying one parameter while fixing all the other parameters, and the results are shown in **Figure S1**. From **Figure S1(a)** we can see that the model tends to be converged with the increment of epoch number. Learning rate controls the step size of gradient descent determining whether the algorithm can obtain its optimal solution or not. A large learning rate will lead to divergence, and a small learning rate may cause convergence slowly. **Figure S1(b)** shows that the optimal learning rate for our model is 0.001. Weight decay factor controls the weights reduction in deep learning model to avoid over-fitting, and the performace influenced by weight decay factor is shown in **Figure S1(c)**. We optimize our model with 2000 epochs, and the initial learning rate and weight decay factor are set as 0.001 and 1.0, respectively.

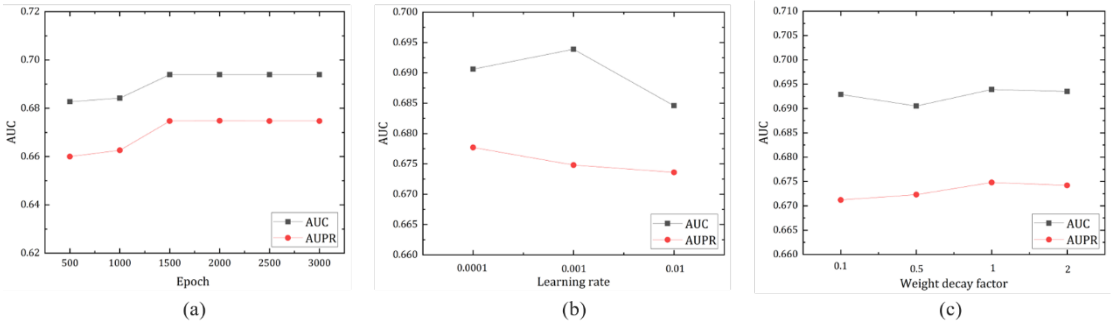

Figure S1. Parameter analysis of iPiDA-GCN. The influence of epoch, learning rate and weight decay factor on the performance of iPiDA-GCN is shown in (a), (b) and (c), respectively.
